# Facile synthesis of size-tunable L-carnosine-capped silver nanoparticles and their role in metal ion sensing and catalytic degradation of *p*-nitrophenol

**DOI:** 10.3762/bjnano.15.124

**Published:** 2024-12-06

**Authors:** Akash Kumar, Ridhima Chadha, Abhishek Das, Nandita Maiti, Rayavarapu Raja Gopal

**Affiliations:** 1 Nanomaterial Toxicology Laboratory, Drug and Chemical Toxicology Group, Food, Drug and Chemical, Environment and Systems Toxicology (FEST) Divison, CSIR-Indian Institute of Toxicology Research (CSIR-IITR), Vishvigyan Bhawan, 31 Mahatma Gandhi Marg, Lucknow 226001, India; 2 Academy of Scientific and Innovative Research (AcSIR), Ghaziabad 201002, India; 3 Radiation & Photochemistry Division, Bhabha Atomic Research Centre, Mumbai-400085, India; 4 Homi Bhabha National Institute, Anushaktinagar, Mumbai-400085, India

**Keywords:** catalysis, heavy metals, ʟ-carnosine, *p*-nitrophenol, silver nanoparticles

## Abstract

ʟ-Carnosine is a dipeptide with notable antioxidant, antiglycation, metal chelating, and neuroprotective properties. Despite its many biological roles, applying ʟ-carnosine as a capping agent in nanoparticle synthesis has remained underexplored. This study explores the potential of ʟ-carnosine in synthesizing tunable plasmonic silver nanoparticles (ʟ-car-AgNPs). The formation of ʟ-car-AgNPs was confirmed via UV–vis optical absorption spectroscopy, showing single and double plasmonic peaks, depending on the synthesis conditions. Physicochemical characterization using TEM, FTIR, and Raman spectroscopy, as well as EDX and XRD revealed controlled aggregation, successful capping, and crystalline growth of the ʟ-car-AgNPs. The ʟ-car-AgNPs exhibited promising sensing capabilities with limits of detection of 141.79 ppb (1.2 μM) for Cd^2+^, 131.33 ppb (0.63 μM) for Pb^2+^, 215.35 ppb (2.8 μM) for As^3+^, and 245.49 ppb (4.7 μM) for Cr^3+^. Additionally, these nanoparticles demonstrated catalytic activity regarding the degradation of *p*-nitrophenol (P-NP), achieving complete degradation of 0.25 and 1 mM solutions within 5 and 10 min, respectively. This study reveals the potential of ʟ-car-AgNPs for both heavy metal ion detection and catalytic degradation of P-NP, indicating their suitability for environmental monitoring and remediation applications. Further optimization and research are needed to expand their environmental applications and to understand their interaction mechanisms with various contaminants.

## Introduction

The persistent rise in environmental pollution, notably from heavy metal ions and organic pollutants, has propelled the development of innovative and efficient environmental monitoring and remediation methods. Five heavy metals, namely, mercury (Hg^2+^), lead (Pb^2+^), cadmium (Cd^2+^), chromium (Cr^3+^), and arsenic (As^3+^), pose severe risks to human health and ecological systems because of their non-biodegradable nature and long biological half-lives, leading to bioaccumulation and biomagnification [1]. Similarly, organic pollutants such as *p*-nitrophenol (P-NP), from agricultural and industrial processes, are of significant concern because of their toxicity and resistance to degradation [2]. Consequently, detection and removal of these contaminants have become crucial for environmental sustainability and public health.

Silver nanoparticles (AgNPs) have attracted the interest of researchers worldwide in recent years because of their promising use in environmental chemistry. The unique optochemical properties of AgNPs, including high surface area to volume ratio, optical absorbance, excellent conductivity, and potent catalytic activity, make them ideal candidates for environmental monitoring and remediation [3]. Modifying silver nanoparticles with various biological molecules, peptides, proteins, and enzymes has further enhanced their functionality, stability, and selectivity towards specific pollutants [4–6]. Biomolecule-capped silver nanoparticles, particularly those stabilized by naturally occurring peptides such as ʟ-carnosine, have shown exceptional sensing and catalytic degradation capabilities. ʟ-Carnosine, a dipeptide consisting of β-alanine and histidine, stabilizes the nanoparticle structure and imparts functional attributes such as metal ion chelation and catalytic enhancement. These features make ʟ-carnosine-capped AgNPs an ideal candidate for environmental applications. It was also reported that pristine AgNPs significantly interact with ʟ-carnosine [7].

Traditional methods for heavy metal ion monitoring in environmental samples involve complex analytical instrumental techniques such as atomic absorption spectroscopy, inductively coupled plasma mass spectrometry, and high-performance liquid chromatography [8–9]. Environmental remediation of P-NP requires processes such as Fenton-like oxidation or ozonation [10]. These techniques require expensive equipment, specialized operational skills, and extensive sample preparation, making them less practical for on-site testing, frequent monitoring, and remediation.

In response to these challenges, recent advancements in nanotechnology have ushered in the development of nanoparticle-based systems that offer promising alternatives because of their simplicity, cost-effectiveness, and potential for real-time applications. Plasmonic nanoparticles, such as silver, have been widely explored for their unique plasmonic and catalytic properties [3,11–12]. These include localized surface plasmon resonance (LSPR), which can be utilized to detect heavy metal ions. The catalytic properties can be applied to degrade nitrophenolic compounds such as P-NP. Also, it is well documented that the properties of silver nanoparticles can be modulated through surface chemistry and other parameters such as size and shape [13]. Kästner and Thünemann described the catalytic degradation of P-NP using silver nanoparticles with the activity depending on the capping agents [14]. To design a dual-functional system for environmental applications, a potential capping and stabilizing agent is required for silver nanoparticle synthesis.

Maiti et al. demonstrated that the dipeptide ʟ-carnosine interacted highly with pristine silver nanospheres [6]. However, its role as a stabilizing/capping agent was never explored for nanoparticle synthesis and advanced environmental applications. Current research has paved the way for developing ʟ-carnosine-capped AgNPs (ʟ-car-AgNPs) enabling environmental monitoring and remediation applications. In addition, ʟ-carnosine is a natural compound widely present in the human brain and meat products. Also, it was reported that grafting magnetic nanoparticles with ʟ-carnosine significantly enhanced the catalytic performance of the nanoparticles [15]. In another study, metal-organic framework nanoparticles fabricated with ʟ-carnosine were employed for arsenic removal via an adsorption mechanism. The maximum removal of arsenic was 94.33 mg/g at a pH of 8.5 and 0.4 g/L adsorbent [16]. These studies confirmed that ʟ-carnosine adsorbed on metal surfaces has widespread environmental applications. However, magnetic nanoparticles or MOFs coated with ʟ-carnosine were applicable only for environmental remediation but were incapable of detecting heavy metal ions. This drawback was overcome by using ʟ-carnosine in combination with silver nanoparticles, as their strong interaction was reported previously. Silver nanoparticles with other capping agents can also be used for either heavy metal sensing or degradation of P-NP. A study explored the potential of gelatine-embedded silver nanoparticles in the degradation of P-NP, but it was not used for heavy metal ion sensing [17]. Similarly, citrate-stabilized silver nanospheres were used for the colorimetric detection of Ni^2+^ with a threshold limit of 0.75 mM without any catalytic activity [18]. Therefore, it was hypothesized that the synthesis of silver nanoparticles capped with ʟ-carnosine may provide two functionalities, that is, monitoring heavy metal ions and degradation of P-NP. Another challenge in sensing heavy metals with capped silver nanoparticles is to detect multiple metals simultaneously via ionic interactions between the exposed functional groups of the capping agent and metal ions. This issue can be addressed using ʟ-carnosine-capped nanosilver because the interaction of ʟ-carnosine with different metal ions has already been reported [19]. The electrochemical detection of Hg^2+^, Pb^2+^, and Cd^2+^ was achieved via ʟ-carnosine–metal interaction [20–21]. With this study, an attempt has been made to address the issues associated with traditional environmental monitoring and remediation systems. This article aims to develop a metal ion sensing and catalyst platform with in situ synthesized ʟ-carnosine-capped silver nanoparticles. The metal ion selectivity (regarding Cd^2+^, Pb^2+^, As^3+^, and Cr^3+^) was monitored via colorimetric and spectrophotometric approaches. Furthermore, the catalytic activity was assessed using P-NP as a reagent. Through this study, we aim to contribute to the advancement of nanoparticle-based technologies to tackle some of the most pressing environmental challenges of our times.

## Materials and Methods

### Materials

ʟ-Carnosine (cat. no. 29825, purity ≥95%), silver nitrate (AgNO_3_, cat. no. 1.93200.0027, purity ≥99.5%), and sodium borohydride (NaBH_4_, cat. no. 480886, purity 99.99%) were obtained from Cayman Chemical, Merck, and Sigma Aldrich, respectively. *p*-Nitrophenol (P-NP, cat. no. 144956, purity 99.5%) and sodium chloride (NaCl, cat. no. 41721, purity 99.9%) were purchased from SRL Chemicals, India. Sodium hydroxide (NaOH, cat. no. TC1460, purity 99.0%), along with a NIST-grade metal standard including arsenic (As^3+^), aluminum (Al^3+^), cadmium (Cd^2+^), zinc (Zn^2+^), mercury (Hg^2+^), nickel (Ni^2+^), copper (Cu^2+^), chromium (Cr^3+^), iron (Fe^3+^), molybdenum (Mo^2+^), and lead (Pb^2+^) were acquired from CDH Fine Chemicals. The chemicals were used without further purification. Before the experiment, glassware was cleaned with aqua regia and rinsed twice with double distilled (DD) water.

### Methods

#### Tunable plasmonic silver nanoparticle synthesis using ʟ-carnosine

Silver nanoparticles with tunable plasmon wavelength were synthesized using a wet-chemical reduction approach. ʟ-Carnosine-capped silver nanoparticles were prepared in the presence of NaBH_4_, a strong reductant. The synthesized ʟ-carnosine-capped silver nanoparticles were named according to their increasing order of plasmon peaks, that is, ʟ-car-AgNP1, ʟ-car-AgNP2, ʟ-car-AgNP3, ʟ-car-AgNP4, and ʟ-car-AgNP5.

ʟ-car-AgNP1 was synthesized at room temperature (RT = 25 °C), with the addition of 1 mL ʟ-carnosine (0.01 M) to 5 mL DD water followed by sequential additions of 100 μL NaOH (1 M), 1 mL AgNO_3_ (0.01 M), and 1 mL NaBH_4_ (0.001 M). The other samples, ʟ-car-AgNP2, ʟ-car-AgNP3, ʟ-car-AgNP4, and ʟ-car-AgNP5, were synthesized at temperatures of 40, 60, and 80 °C and at boiling temperature, respectively. The synthesis procedure for ʟ-car-AgNP2 to ʟ-car-AgNP5 was similar, with the reaction mixture comprising 1 mL ʟ-carnosine (0.01 M) added to 5 mL DD water, followed by sequential additions of 50 μL NaOH (1 M), 1 mL AgNO_3_ (0.01 M), and 2 mL NaBH_4_ (0.001 M). The term “ʟ-car-AgNPs” is used throughout the article to refer to the synthesized silver nanoparticles collectively. Furthermore, nanopowders of the samples were prepared using 1 M NaCl solution via the precipitation method, and the obtained precipitate was air-dried.

#### Characterization of ʟ-carnosine-capped silver nanoparticles

The spectral absorbance of ʟ-car-AgNPs was recorded using an Epoch2 spectrophotometer (BioTek, USA) in the 300–900 nm range. Zeta potentials and average hydrodynamic diameters of ʟ-car-AgNPs were determined using a Zetasizer (Nano ZS, Malvern, UK). The hydrodynamic size of ʟ-car-AgNPs was measured by placing them in 1 mL disposable cuvettes (DTS0012), while the zeta potential was measured using zeta cuvettes (ZEN1020). The ʟ-car-AgNPs samples were observed under a transmission electron microscope (TEM, 120 kV, FEI Tecnai, Netherlands) for a detailed examination of size and morphology. The crystalline structure of ʟ-car-AgNP1 was determined using X-ray diffraction (XRD, Rigaku Smartlab, Japan) within a 2θ range of 35° to 80°. Nanoparticle solutions were air-dried, and the obtained nanopowders (20 mg) were used for measurements. The functionalization of AgNPs with ʟ-carnosine was validated through Fourier-transform infrared spectroscopy (FTIR, Thermo Scientific Nicolet iS5, USA). For this, 10 mg of air-dried nanopowder was placed over a diamond, and the software (OMNIC™) was run over the spectral range of 400–4000 cm^−1^.

The Raman spectra of ʟ-carnosine-capped silver nanoparticles, as well as ʟ-carnosine, as solid and in aqueous solution, were recorded at RT using an excitation source of 532 nm. The light source was a diode-pumped solid-state Nd^3+^:YAG laser (Cobolt Samba 0532-01-0500-500, Cobolt AB, Sweden). For solid ʟ-carnosine and ʟ-car-AgNPs, powdered samples were placed on glass slides, and the Raman signal was collected in a backscattering geometry using a 50× long working distance objective. The incident laser of approximately 10 mW power had a spot size of approximately 50 μm on the sample surface. Aqueous ʟ-carnosine solutions were analyzed using a standard cuvette (1 cm), and the Raman-scattered light was collected at an angle of 180°. The Raman signal detection was achieved with a charge-coupled device (CCD, Synapse, Horiba Jobin Yvon) attached to a monochromator (LabRAM HR800, Horiba Jobin Yvon, France) and edge filter using 532 nm as an excitation source. The Raman signal of the silicon wafer at 520 cm^−1^ was considered the reference point for spectrometer calibration, ensuring a spectral measurement accuracy better than 1 cm^−1^. The Raman spectra acquisition times were approximately 25 s for solid ʟ-carnosine and ʟ-car-AgNPs and 100 s for ʟ-carnosine in aqueous solution.

#### DFT calculations

Theoretical insights into the FTIR and Raman measurements were obtained through molecular structure optimization of ʟ-carnosine, its anionic form, and probable ʟ-carnosine–(Ag)_4_ complexes. The structure optimization was performed in vaccum and water as solvent using the SCRF (CPCM) model to account for the effect of the environment. The computations were performed using density functional theory (DFT) with the B3LYP functional [22] and split basis sets 6-31+G* for all atoms (C, O, N, and H), and LANL2DZ for Ag atoms. Calculations were executed using Gaussian 09, Revision A.02 software [23]. The optimization process was conducted without symmetry restrictions. The convergence criteria were an energy change of less than 1.0 × 10^−6^ Hartree and a gradient of less than 3.0 × 10^−4^ atomic units. The vibrational frequencies of ʟ-carnosine, its anion, and the probable Ag_4_ complexes were computed at the optimized geometries. Subsequently, the theoretical vibrational spectra were compared with the experimental Raman spectra of ʟ-carnosine in various forms, namely, in the solid state, in aqueous solution, in alkaline solution, and as capping agents in silver nanoparticles (ʟ-car-AgNPs).

#### Sensing of heavy metals

Metal standards (NIST grade, 1000 ppm) of As^3+^, Al^3+^, Cd^2+^, Zn^2+^, Hg^2+^, Ni^2+^, Cu^2+^, Cr^3+^, Pb^2+^, Mo^2+^, and Fe^3+^ were diluted to a concentration of 10 ppm. A uniform concentration of nanoparticles in terms of optical density (OD) was maintained throughout the experiment. The ʟ-car-AgNPs were fixed at an optical density of 1 ± 0.2 OD for the sensing experiment. The final volume of ʟ-car-AgNPs and the corresponding metal ions was 1 mL. In a typical sensing experiment, the metal ions were spiked in water to an end concentration of 0.5 ppm, followed by the addition of ʟ-car-AgNPs as a sensing probe. The dispersion was then examined for colorimetric/spectrophotometric changes. Finally, the limit of detection (LOD) and the limit of quantification (LOQ) of the developed nanosensors were estimated through a linear plot of the absorption as a function of the concentration of the detected metal ions.

#### ʟ-carnosine-capped silver nanoparticles as the catalytic agent

ʟ-car-AgNPs were evaluated regarding their catalytic performance in the degradation of P-NP as a model substrate. P-NP is reduced and forms *p*-aminophenol (P-AP) in the presence of nanoparticle catalyst and NaBH_4_. All catalysis experiments were performed at RT in a 3.5 mL quartz cuvette. The reagents were introduced in the sequence of 2 mL P-NP (0.25 mM or 1 mM), 1 mL of NaBH_4_ (100 mM), and 50 μL of ʟ-carnosine-capped AgNPs (1 ± 0.2 OD). The degradation of P-NP and the formation of P-AP were monitored by recording UV–vis spectra in the 300–900 nm range. The disappearance of the yellow color, characteristic of the nitrophenolate ion, was observed upon the addition of the AgNP catalyst, accompanied by a concomitant decrease in the absorbance intensity. The catalytic efficiency was quantified using the following equation:




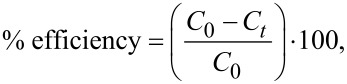




where *C*_0_ represents the initial concentration at *t* = 0 and *C*_t_ denotes the concentration at time *t*.

## Results and Discussion

### Synthesis of ʟ-carnosine-capped tunable silver nanoparticles

**Figure 1:**
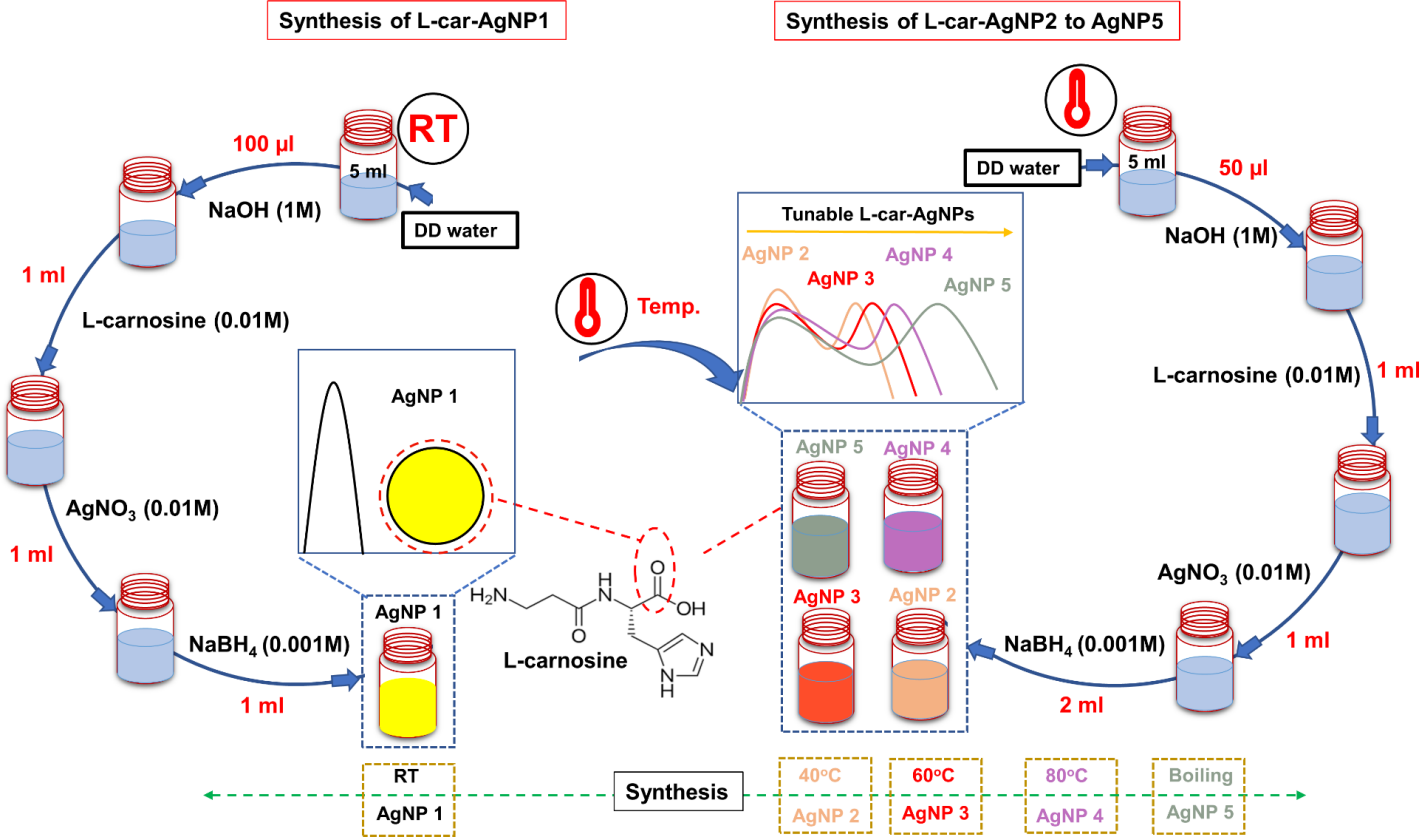
Schematic representation of the synthesis of tunable ʟ-carnosine-capped silver nanoparticles (ʟ-car-AgNPs) along with the critical parameters. The samples ʟ-car-AgNP1, AgNP2, AgNP3, AgNP4, and AgNP5 were synthesized at RT, 40, 60, and 80 °C and at boiling temperature, respectively.

ʟ-car-AgNPs were synthesized using a wet-chemical reduction approach. The precursor metal salt was reduced in the presence of the stabilizing or capping agent. Figure 1 shows a schematic representation of the ʟ-car-AgNP synthesis. The sample ʟ-car-AgNP1 was synthesized at room temperature by sequentially mixing water, NaOH, ʟ-carnosine, AgNO_3_, and NaBH_4_. The obtained nanoparticles were bright yellow in color, confirming their spherical shape, as reported in previous studies. For the synthesis of the tunable ʟ-car-AgNP2 to ʟ-car-AgNP5 samples, a slightly modified protocol was used compared to ʟ-car-AgNP1. The modifications in terms of temperature and the ratio of NaBH_4_ to NaOH led to the formation of ʟ-car-AgNPs with different colors. The color of the obtained nanoparticles suggested that they were in a controlled aggregated state. Further characterization was performed to analyze the physicochemical properties of the synthesized ʟ-carnosine-capped silver nanoparticles.

### Optical spectroscopy of ʟ-carnosine-capped silver nanoparticles

**Figure 2:**
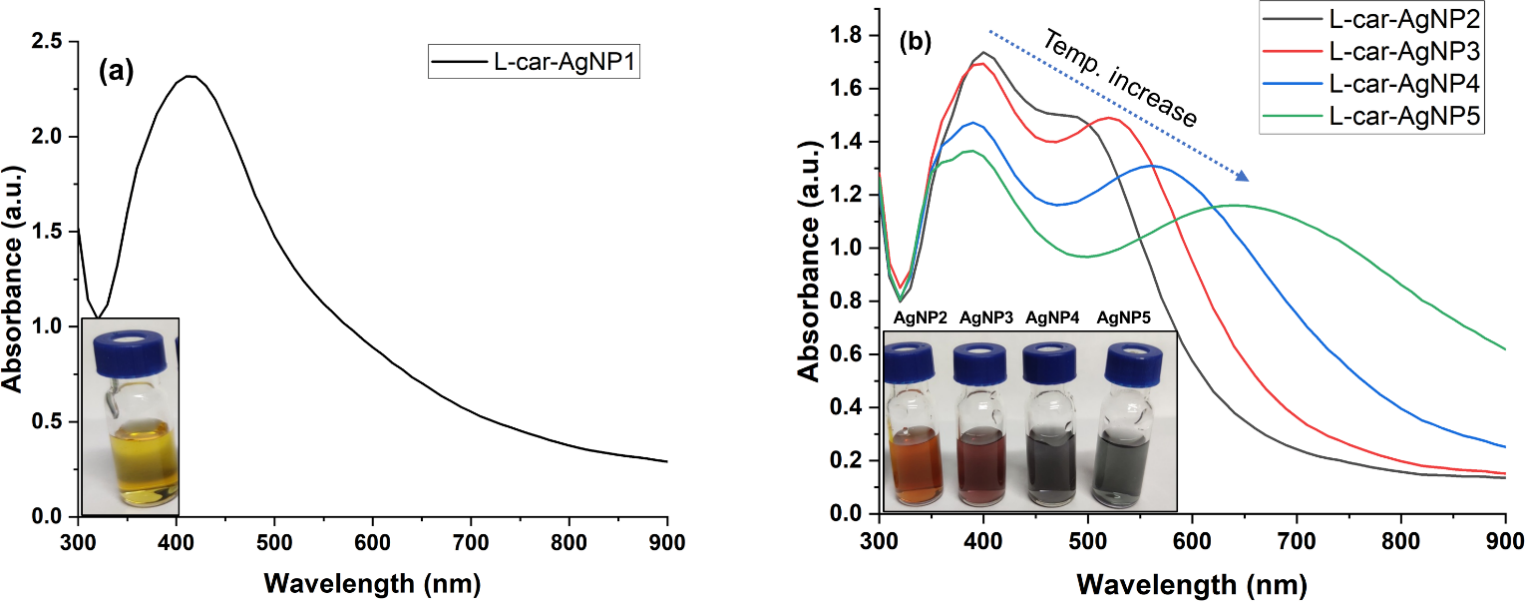
Optical absorbance of (a) ʟ-carnosine-capped AgNP1 and (b) ʟ-carnosine-capped AgNP2–AgNP5. The insets of the figure show photographs of the synthesized nanoparticle suspensions.

**Figure 3:**
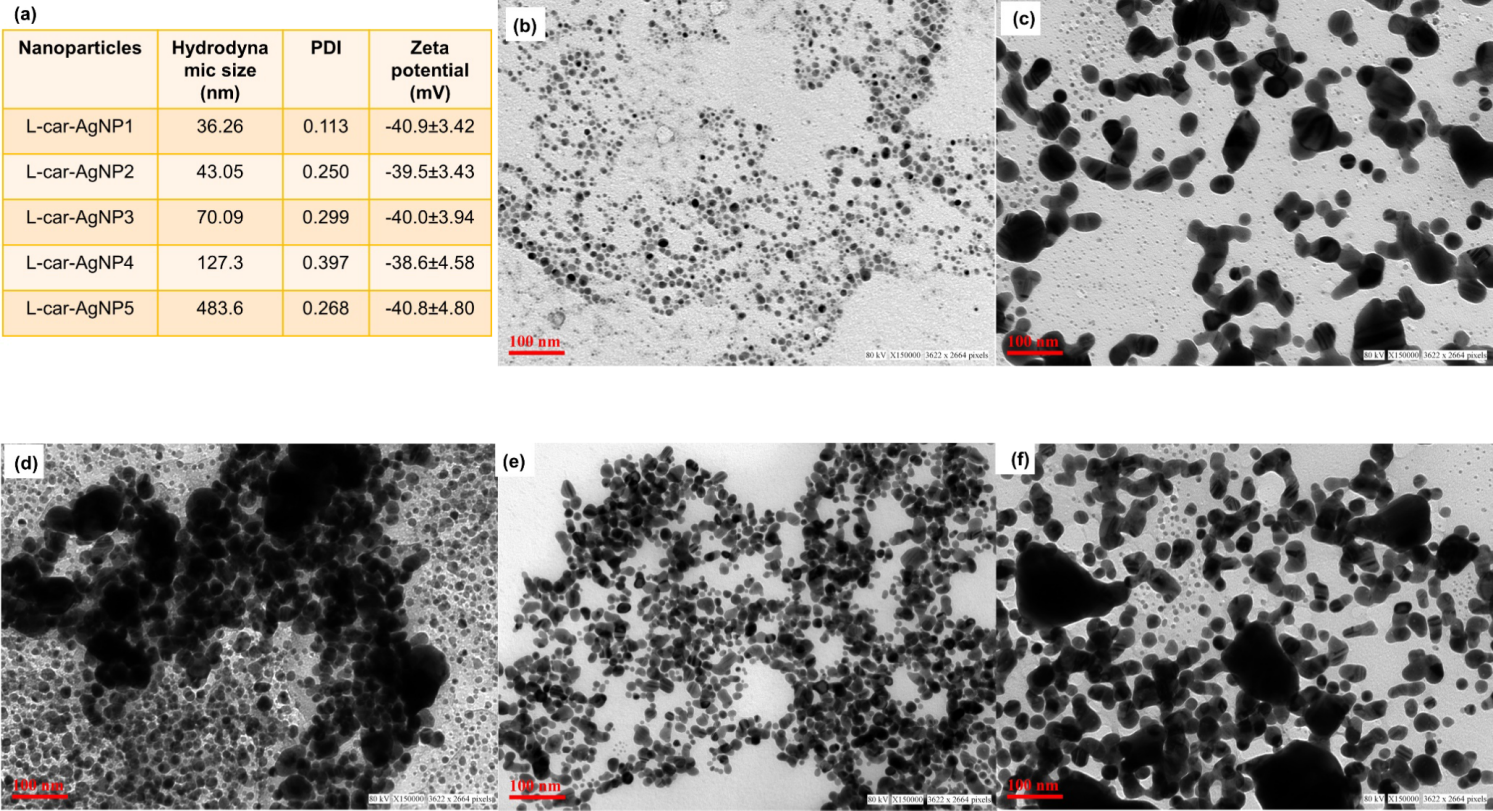
(a) Hydrodynamic size and zeta potential of ʟ-carnosine-capped silver nanoparticles and TEM micrographs of (b) ʟ-car-AgNP1, (c) ʟ-car-AgNP2, (d) ʟ-car-AgNP3, (e) ʟ-car-AgNP4, and (f) ʟ-car-AgNP5.

The synthesized silver nanoparticles capped with ʟ-carnosine were assessed regarding their absorption peaks, as illustrated in Figure 2. The sample ʟ-car-AgNP1 exhibited a single absorption peak at 410 nm (Figure 2a). All other samples displayed two absorption peaks, that is, the first near 400 nm and the second at 500 nm (ʟ-car-AgNP2), 530 nm (ʟ-car-AgNP3), 590 nm (ʟ-car-AgNP4), and 650 nm (ʟ-car-AgNP5) (Figure 2b). The observed absorption bands are attributed to LSPR. This phenomenon is an intrinsic property of noble metal nanoparticles [3]. The inset in Figure 2 shows a change in color from bright yellow (ʟ-car-AgNP1) to dark yellow, ruby red, blue, and blackish blue for ʟ-car-AgNP2, AgNP3, AgNP4, and AgNP5, respectively. The sample ʟ-car-AgNP1 shows a single absorption peak and a bright yellow color, which confirms that the nanoparticles are spherical and monodispersed. However, silver nanoparticles with anisotropic shape were reported to have two absorption bands, namely transverse and longitudinal [24]. Furthermore, the color of the anisotropic nanosilver is different from yellow, which is also observed in the samples ʟ-car-AgNP2 to ʟ-car-AgNP5. The transverse and longitudinal absorption bands might be correlated to the formation of polydisperse nanoparticles prepared at higher temperatures because it has been reported that Ostwald ripening occurs faster at higher temperatures [25]. Therefore, it is assumed that the higher temperature, along with the ratio of NaOH to NaBH_4_, led to the formation of aggregated ʟ-car-AgNPs. These results contradict earlier reports and confirm the formation of aggregated ʟ-car-AgNPs with two absorption bands. However, despite the different shapes, absorption bands and colors of the ʟ-car-AgNPs samples are similar to those published on CTAB-capped silver nanorods [24]. The formation of aggregated nanoparticles in the current study is probably due to the capping of ʟ-carnosine instead of CTAB. In addition, ʟ-carnosine induces a second absorption peak in the pristine silver nanospheres upon interaction. The appearance of the redshifted peak might be due to charge transfer or aggregation [7]. An increased nanoparticle size leads to a further redshift of the plasmonic peak [26]. Hydrodynamic size, zeta potential, and morphology of the ʟ-car-AgNPs are shown in Figure 3.

Information on average hydrodynamic size and distribution of nanoparticles in colloidal solution was obtained from DLS measurements. The hydrodynamic sizes of ʟ-car-AgNP1, ʟ-car-AgNP2, ʟ-car-AgNP3, ʟ-car-AgNP4, and ʟ-car-AgNP5 were 36.26, 43.05, 70.09, 127.3, and 483.6 nm, respectively (Figure 3a and Supporting Information File 1, Figure S1). The sequential increase in nanoparticle size confirms nanoparticle aggregation or formation of bigger particles and is consistent with the absorption spectra in Figure 2.

The polydispersity index (PDI) is a dimensionless value that measures the width of the size distribution of particles in a sample. The PDI values of ʟ-car-AgNP1, ʟ-car-AgNP2, ʟ-car-AgNP3, ʟ-car-AgNP4, and ʟ-car-AgNP5 were 0.113, 0.250, 0.299, 0.397, and 0.268, respectively (Figure 3a). A PDI value below 0.3 typically indicates a relatively narrow and well-controlled size distribution [27]. ʟ-Carnosine forms a monolayer around the nanoparticles, providing a consistent and uniform surface coverage. This uniformity in surface passivation contributes to the narrow size distribution of the nanoparticles. The formation of stable silver nanoparticles capped with ʟ-carnosine was confirmed via zeta potential measurements. The zeta potential values of ʟ-car-AgNP1, ʟ-car-AgNP2, ʟ-car-AgNP3, ʟ-car-AgNP4, and ʟ-car-AgNP5 were −40.9 ± 3.42, −39.5 ± 3.43, −40.9 ± 3.94, −38.6 ± 4.58, and −40.9 ± 4.80, respectively. Zeta potentials beyond ±30 mV indicate excellent colloidal stability due to strong repulsive forces among the nanoparticles (Figure 3a and Supporting Information File 1, Figure S2) [28]. The highly negative zeta potential values are probably due to the exposed functional group of ʟ-carnosine molecules. The negatively charged carboxyl groups (COO^−^) of ʟ-carnosine are attracted towards the positively charged surfaces of silver.

The morphology and size of silver nanoparticles capped with ʟ-carnosine were measured using TEM (Figure 3b–f). The TEM micrograph of ʟ-car-AgNP1 (Figure 3b) indicates the formation of spherical particles with high monodispersity and is consistent with the optical absorbance and DLS measurements. However, ʟ-car-AgNP2–5 (Figure 3c–f) show ʟ-carnosine-induced controlled aggregation (formation of bigger size particles) upon increasing the temperature, which is also consistent with the optical absorption and DLS measurements. The micrograph of samples with two absorption peaks indicates that the nanoparticles aggregated. The results confirm that ʟ-car-AgNP2–5 nanoparticles are polydisperse.

### FTIR, Raman spectroscopy and X-ray diffraction

**Figure 4:**
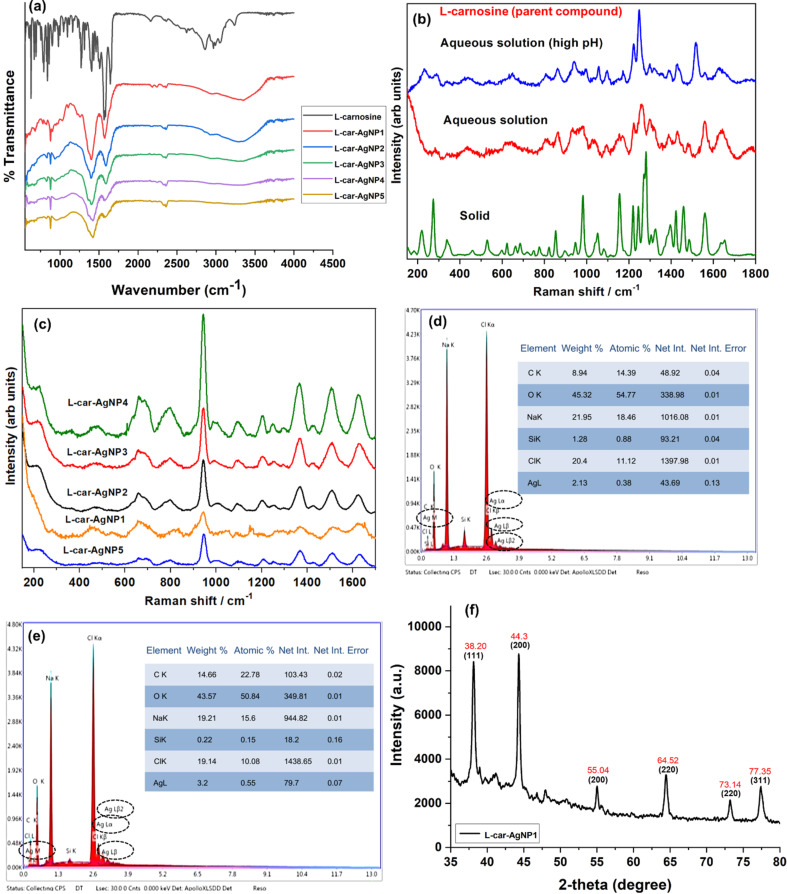
(a) FTIR spectra of ʟ-carnosine and ʟ-car-AgNPs. Raman spectra of (b) ʟ-carnosine (solid and aqueous solution) and (c) ʟ-car-AgNPs. SEM-EDX data confirms the presence of silver in (d) ʟ-car-AgNP1 and (e) ʟ-car-AgNP2. (f) XRD pattern of ʟ-carnosine AgNP1.

In addition to TEM analysis, the interaction between functional groups of ʟ-carnosine and silver, the elemental composition, and the diffraction pattern of ʟ-car-AgNPs were assessed using FTIR, Raman, EDX, and XRD, as shown in Figure 4.

Figure 4a shows the FTIR analysis of pure ʟ-carnosine and its interaction with the silver nanoparticles in ʟ-car-AgNPs. In the case of pure ʟ-carnosine, a characteristic NH_2_ asymmetric stretching frequency at ca. 3243 cm^−1^ overlaps with the broad OH peak of H_2_O. The imidazole (C=C) stretching and carboxylate (COO^−^) asymmetric stretching frequencies are observed at 1568 and 1404 cm^−1^, respectively [7]. However, in the case of ʟ-car-AgNPs, the peak at 3243 cm^−1^ disappeared, which might be due to the interaction of the NH_2_ groups with silver nanoparticles or their proximity to the nanoparticles’ surface. Several prominent overlapping peaks were observed at 1576 cm^−1^ (imidazole C=C stretching), 1404 cm^−1^ (carboxylate, COO^−^ asymmetric stretching), and 878 cm^−1^ (asymmetric NH_2_ wagging) in ʟ-car-AgNPs. This suggests that the nanoparticles were capped with ʟ-carnosine.

Figure 4b shows the Raman spectrum of pure ʟ-carnosine in solid and aqueous phases at normal and alkaline pH values. The prominent Raman bands in solid ʟ-carnosine are observed at 3204 cm^−1^ (symmetric NH_2_ stretching), 3082 cm^−1^ (imidazole (imz) ring CH stretching), 2906 cm^−1^ (symmetric CH_2_ stretching), 1560 cm^−1^ (imz ring C=C stretching), 1458 cm^−1^ (imz ring C–N stretching), 1422 cm^−1^ (amide NH bend), 1281 cm^−1^ (imz ring breathing), 1219 cm^−1^ (CH_2_ twist), 1156 cm^−1^ (NH_2_ twist), 1051 cm^−1^ (CH_2_ rock), 982 cm^−1^ (imz ring deformation), 854 cm^−1^ (NH_2_ wag), 623 cm^−1^ (amide NH out of plane bend), 528 cm^−1^ (amide NCO bend), 339 cm^−1^ (CO_2_ bend) and 273 cm^−1^ (imz ring rotation). The Raman spectra of a saturated solution of ʟ-carnosine at normal (and alkaline) pH shown in Figure 4b display strong peaks at 3265 (3266), 2903 (2909), 1639 (1628), 1560 (1577), 1481 (1516), 1428 (1428), 1298 (1299), 1260 (1249), 1223 (1222), 1037 (1057), 981 (997), 935 (941), and 863 (862) cm^−1^, which are assigned to symmetric NH_2_ stretching, symmetric CH_2_ stretching, amide C=O stretching, imz ring C=C stretching, CH_2_ scissoring, amide NH bend, imz ring breathing, imz ring C–N stretching, CH_2_ twist, CH_2_ rock, imz ring deformation, C–CO_2_ stretching, and NH_2_ wagging, respectively. The symmetric CO_2_ stretching vibration in ʟ-carnosine at normal (alkaline) pH appears weakly at 1388 (1391) cm^−1^. The observed shift in the Raman bands of ʟ-carnosine in aqueous solution compared to those in the solid is probably due to solvent effects.

Figure 4c shows surface-enhanced Raman spectra (SERS) of ʟ-carnosine-capped silver nanoparticles. The signal-to-noise (S/N) ratio follows the order ʟ-car-AgNP4 > ʟ-car-AgNP3 > ʟ-car-AgNP2 > ʟ-car-AgNP1 > ʟ-car-AgNP5. Therefore, the results show maximum SERS enhancement for AgNP4. This is probably because of the greater surface roughness of the nanoparticles, leading to maximum electromagnetic enhancement in AgNP4. The SERS spectra of the ʟ-car-AgNPs are almost similar and show Raman bands at 1623 cm^−1^ (amide C=O stretching), 1505 cm^−1^ (imz ring C=C stretching), 1365 cm^−1^ (CO_2_ symmetric stretching), 1204 cm^−1^ (CH_2_ twist), 1092 cm^−1^ (C–NH_2_ stretching), 994 cm^−1^ (imz ring deformation), 943 cm^−1^ (C-CO_2_ stretching), 792 cm^−1^ (imz ring NH bend), 685 cm^−1^ (CO_2_ bend), 660 cm^−1^ (amide NH bend), 473 cm^−1^ (NH_2_ twist) and 215 cm^−1^ (Ag–O stretching). The observation of the abovementioned prominent peaks in the SERS spectra confirms the capping of silver nanoparticles with ʟ-carnosine. The SERS enhancement observed in various vibrations associated with imidazole ring, CO_2_, and NH_2_ groups suggests that, at alkaline pH, the anionic form of ʟ-carnosine is bound to the AgNPs surface through the carboxylate group and the imidazole as well as amino groups remain close to the silver nanoparticles’ surface. The optimized structure of the ʟ-carnosine–(Ag)_4_ complex (Supporting Information File 1, Figure S4c) supports the experimental results well.

FTIR and Raman spectroscopy confirm the successful capping of silver nanoparticles with ʟ-carnosine. The presence of Ag in ʟ-carnosine-capped AgNPs was also assessed using SEM-EDX. Figure 4d and Figure 4e confirm that Ag is present in ʟ-car-AgNP1 and ʟ-car-AgNP2, respectively. The insets of Figure 4d and Figure 4e indicate 2.13 and 3.2 wt % of silver in ʟ-car-AgNP1 and ʟ-car-AgNP2, respectively. However, apart from silver, high fractions of sodium and chlorine were observed. This is probably due to the preparation of the nanopowders using the precipitation method with NaCl. In addition, NaOH used in the synthesis process acts as a source of sodium in the EDX spectrum.

Crystalline structures of ʟ-car-AgNPs were analyzed using X-ray diffraction. The XRD pattern of one sample representative of all synthesized ʟ-carnosine-capped silver nanospheres is given here (Figure 4f). There are prominent peaks at 38.2°, 44.3°, 64.5°, and 77.4°, attribtuted to the (111), (200), (220), and (311) crystal planes, respectively, of face-centered cubic silver. These results are consistent with [29]. However, two minor peaks are also observed at 55.04° and 73.14°, which have also been observed in another study regarding nanoparticles. The peaks are attributed to the (200) and (220) planes of silver oxide [30]. The characterization of ʟ-car-AgNPs confirms that ʟ-carnosine caps the silver nanoparticles and, thus, can be utilized as a sensing and catalytic agent against environmental contaminants.

### DFT results

The optimized geometries of ʟ-carnosine are shown in Supporting Information File 1, Figure S3. ʟ-Carnosine may exist in two H-bonded forms (Supporting Information File 1, Figure S3a,b) where the carboxylic acid and amino groups remain close, and the O–H moiety of the carboxyl group forms a H-bond with the N atom of the amino group. ʟ-Carnosine may also exist in forms where the carboxylic acid and amino groups are far apart (Supporting Information File 1, Figure S3c,d). From the optimized energies, it is observed that the conformation in Figure S3a is the most stable, followed by the ones in Figure S3b, Figure S3c, and Figure S3d, with relative stabilization energies of 0, 1.093, 4.779, and 5.473 kcal/mol, respectively. The optimized geometries of the anionic form of ʟ-carnosine in water are shown in Supporting Information File 1, Figure S4a,b. The optimized energies of the two forms suggest that the form in Figure S4a is more stable than the form in Figure S4b by 2.242 kcal/mol. The optimized geometries of the ʟ-carnosine–(Ag)_4_ complexes are shown in Figure S4c,d. The optimized energies of the ʟ-carnosine–(Ag)_4_ complexes suggest that the form in Figure S4c is more stable than the one in Figure S4d by 1.713 kcal/mol. The infrared and Raman frequencies were computed for all possible forms of ʟ-carnosine, its anionic form, and the ʟ-carnosine–(Ag)_4_ complexes. The observed FTIR and Raman peaks are assigned based on the theoretical (B3LYP/6-31+G*)-computed frequencies.

### Detection of Cd^2+^and Pb^+2^ using ʟ-car-AgNP1

**Figure 5:**
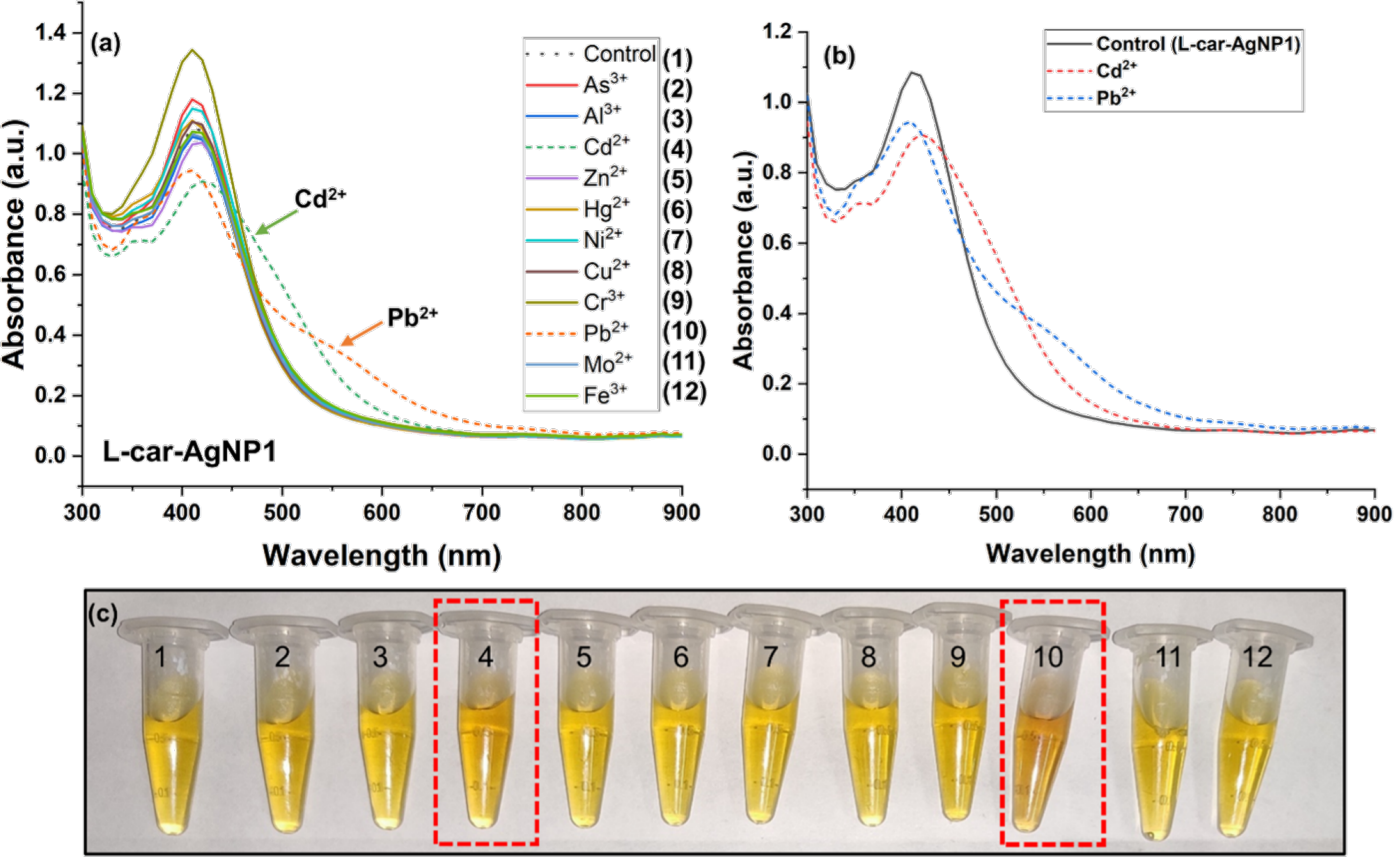
Heavy metal ion sensing using ʟ-car-AgNP1. (a) UV–vis spectrophotometric determination of Cd^2+^, Pb^2+^, and the other metal ions. (b) Optical absorbance of ʟ-car-AgNP1 with and without Cd^2+^ and Pb^2+^. (c) Colorimetric detection of Cd^2+^ and Pb^2+^ using ʟ-car-AgNP1 compared to control (no. 1, without metal ions). The dashed line and the red dashed boxes show the spectrophotometric and colorimetric change upon adding Cd^2+^ (no. 4) and Pb^2+^ (no. 10).

The ʟ-carnosine-capped AgNPs were evaluated regarding the detection of heavy metals via UV–vis spectrophotometry. A series of metal ions, namely As^3+^, Al^3+^, Cd^2+^, Zn^2+^, Hg^2+^, Ni^2+^, Cu^2+^, Cr^3+^, Pb^2+^, Mo^2+^, and Fe^3+^, was used for the sensing experiment. Figure 5 shows the absorption spectra of ʟ-car-AgNP1 mixed with various metal ions. The concentration of ʟ-car-AgNP1 was fixed in terms of optical density at 1 ± 0.2. Figure 5a shows the optical absorbance spectra of ʟ-car-AgNP1 after adding Cd^2+^ and Pb^2+^ and all other metals at a concentration of 0.5 ppm. Figure 5b confirms the specificity of ʟ-car-AgNP1 towards Cd^2+^ and Pb^2+^ compared to the control. The plasmon peak with λ_max_ = 410 nm gradually decreases upon adding Cd^2+^ and Pb^2+^ ions. This is accompanied by a redshift of the absorbance wavelength or the formation of a new peak. Also, ʟ-car-AgNP1 shows a significant color change (yellow to brownish-yellow) in the presence of Cd^2+^ and Pb^2+^ (Figure 5c). The color and absorption spectra change can be attributed to the nanoparticles’ aggregation [31]. A slight increase in the intensity of ʟ-car-AgNPs after the addition of Cr^3+^ ions was also observed, possibly due to the metal–AgNP complex that decreases the interparticle distance followed by a plasmonic and colorimetric change [32]. The ʟ-carnosine structure has amine or carboxyl functional groups. However, zeta potential measurements confirmed that ʟ-car-AgNPs are negatively charged, and the overlapping peaks in FTIR suggested that carboxyl is the exposed functional group. Based on the earlier reports, free –NH_2_ and carboxyl groups are believed to participate in cross-linking and forming complexes between ʟ-car-AgNP1 and Cd^2+^/Pb^2+^ ions. The Cd^2+^ and Pb^2+^ ions interact with lone electron pairs of these exposed groups. We hypothesize that the complexation between ʟ-car-AgNP1 and Cd^2+^/Pb^2+^ decreases the distance between the nanoparticles. This results in a redshift and the emergence of shoulder peaks in the absorption spectra, followed by a decrease in absorption intensity compared to the control. Abate et al. showed that ʟ-carnosine can interact with bivalent ions, including Cd^2+^ [19]. This study also confirms that the bivalent metal ions Pb^2+^ and Cd^2+^ prominently interact with ʟ-car-AgNP1.

### Detection of As^3+^ and Cr^3+,^ using ʟ-car-AgNP2 and ʟ-car-AgNP5

**Figure 6:**
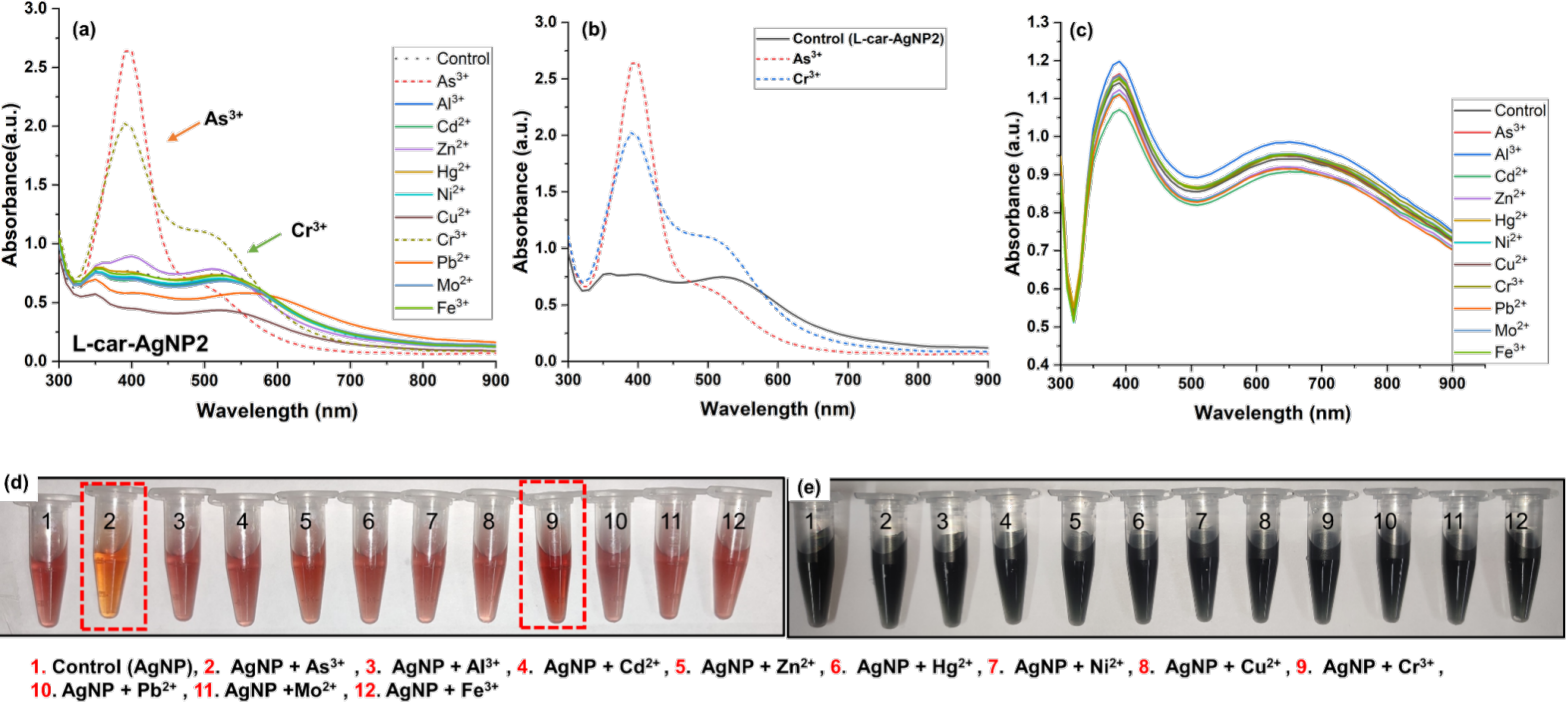
Heavy metal ion sensing using ʟ-car-AgNP2 and ʟ-car-AgNP5. (a) UV–vis spectrophotometric determination of As^3+^ and Cr^3+^ (marked by arrows). (b) Optical absorbance of ʟ-car-AgNP2 with and without As^3+^ and Cr^3+^. (c) Optical spectrometry confirmed no metal ion interaction with ʟ-car-AgNP5. (d) Colorimetric detection of As^3+^ and Cr^3+^ using ʟ-car-AgNP2 compared to the control without metal ions. (e) No colorimetric change was observed in ʟ-car-AgNP5 with or without the metal ions. The dashed lines and the red dashed boxes show the spectrophotometric and colorimetric change upon adding As^3+^ and Cr^3+^.

As^3+^ and Cr^3+^ were detected using ʟ-car-AgNPs that showed a dual absorption bands (transverse and longitudinal). The samples ʟ-car-AgNP2, ʟ-car-AgNP3, and ʟ-car-AgNP4 could detect As^3+^ and Cr^3+^ via colorimetric and spectrophotometric observations (Figure 6a,b,d and Supporting Information File 1, Figure S5). In contrast, ʟ-car-AgNP5 did not detect any metal ions, which might be due to the formation of larger aggregates of silver nanoparticles (Figure 6c). ʟ-car-AgNP2 exhibited a prominent colorimetric and spectrophotometric sensing ability towards As^3+^ and Cr^3+^ (Figure 6a). The concentration of ʟ-car-AgNP2 was fixed at an optical density of 1 ± 0.2, and the concentration of added metal ions was 0.5 ppm. The results show that only the tubes containing As^3+^ and Cr^3+^ display a prominent color change from ruby red (control) to dark yellow (Figure 6d). This implies that ʟ-car-AgNP2 has strong selectivity towards As^3+^ and Cr^3+^. Figure 6b shows the UV–vis absorption spectra of ʟ-car-AgNP2 without metal ions and with As^3+^ and Cr^3+^. The absorption curves reveal that As^3+^ and Cr^3+^ lead to hypsochromic (530 nm) and hyperchromic (400 nm) shifts, respectively, of the plasmon peak. The interaction between negatively charged ʟ-car-AgNP2 and positively charged As^3+^ and Cr^3+^ leads to changes in AgNPs depending on the metal ion concentration, resulting in the deformation of anisotopic to isotropic particles. A similar plasmonic shift has been observed in the literature, where As^3+^ induced the formation of spherical silver nanoparticles after being added to silver nanoprisms [33]. In addition, negatively charged silver nanoparticles are prone to interaction with As^3+^ and Cr^3+^ under basic conditions (pH 10). Earlier reports have confirmed the interaction of Cr^3+^ with metal nanoparticles at basic pH [34]. Therefore, it is assumed that ʟ-car-AgNP2, ʟ-car-AgNP3, and ʟ-car-AgNP4 can stabilize the AgNPs under alkaline conditions (pH 10) and can detect As^3+^ and Cr^3+^ in all samples. This is due to the interaction of As^3+^ and Cr^3+^ with the exposed functional groups on the surface of the silver nanoparticles. In the case of ʟ-car-AgNP5, the nanoparticles lose the ability to interact with the trivalent metal ions because of the formation of bigger particles, which was confirmed by TEM and DLS measurements.

### Interference study and quantification of the detection limit for Cd^2+^ and Pb^2+^ using ʟ-car-AgNP1

**Figure 7:**
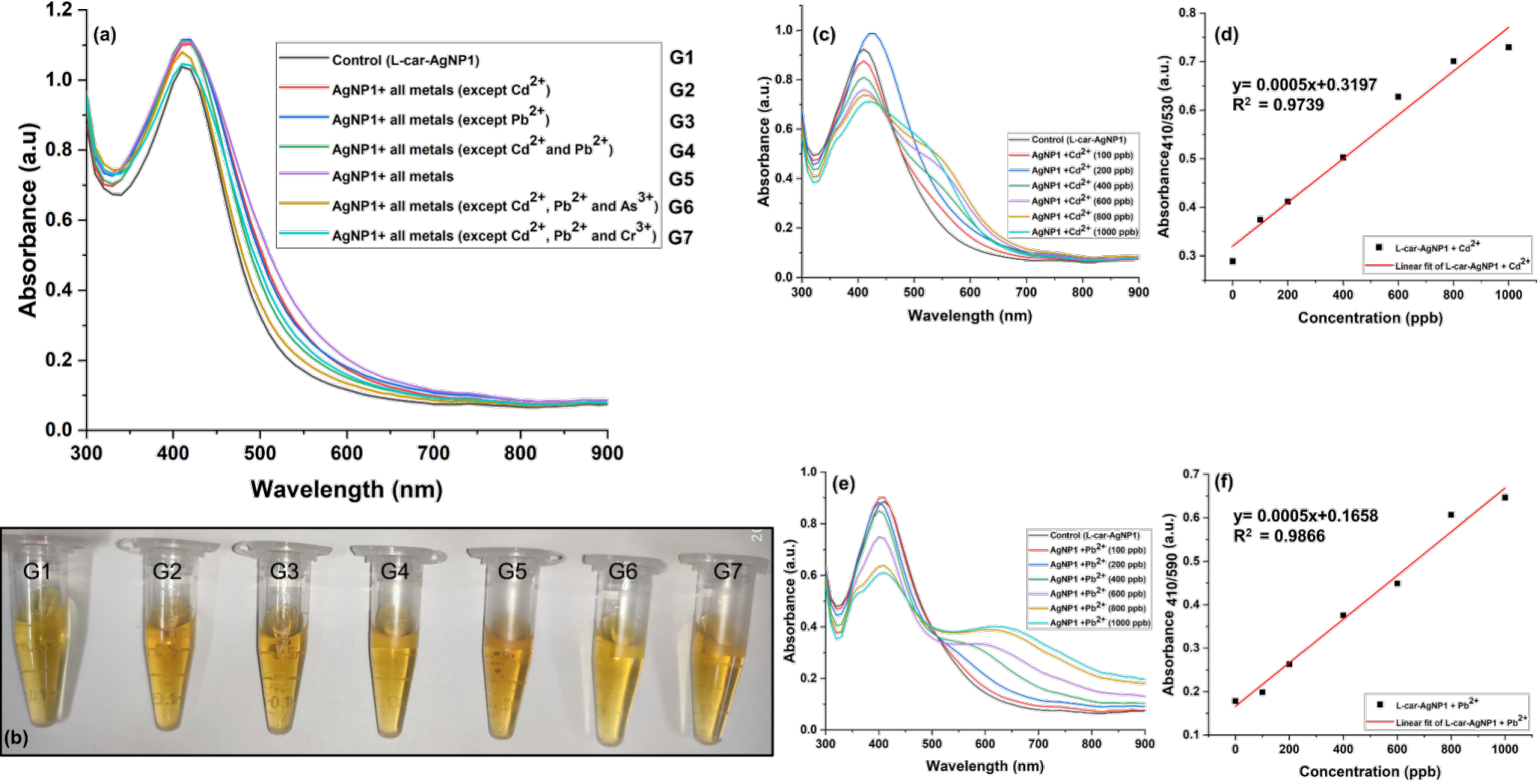
Interference study regarding the metal ion selectivity of ʟ-car-AgNP1. (a) Optical absorbance of different groups 2–7 (G2–G7) and the control sample (G1). (b) Colorimetric observation of metal ion selectivity in the presence of groups 1–7. (c) UV–vis spectral absorbance of ʟ-car-AgNP1 with different Cd^2+^ concentrations (100–1000 ppb) and (d) the corresponding linear curve. (e) UV–vis spectral absorbance of ʟ-car-AgNP1 with different Pb^2+^ concentrations (100–1000 ppb) and (f) the corresponding linear curve.

The ability of ʟ-car-AgNP1 as a probe for the selective detection of Cd^2+^ and Pb^2+^ in the presence of multiple metal ions was assessed using an interference study, where several groups of metal mixtures were prepared (Figure 7). The metal ions, namely As^3+^, Al^3+^, Zn^2+^, Hg^2+^, Ni^2+^, Cu^2+^, Cr^3+^, Mo^2+^, and Fe^3+^, were mixed in a volume of 1 mL with a final concentration of 0.5 ppm for each metal. The metal ion mixtures were grouped as groups G1–7, where G1 included no metal ions (control group). G2 and G3 included all metal ions except either Cd^2+^ or Pb^2+^, respectively. G4 included all metal ions except both Cd^2+^ and Pb^2+^. G5 contained all metal ions including Cd^2+^ and Pb^2+^. After adequate mixing of the metal ions, ʟ-car-AgNP1 was dispersed into the test system and underwent colorimetric and spectrophotometric investigations. The slight color changes in G2, G3, and G5 indicate the detection of Cd^2+^ and Pb^2+^ even when mixed with other metal ions (Figure 7a). In G2, a slight increase and shift in peak intensity in the presence of different metals (λ_max_ = 420 nm, 1.11 OD) compared to the control (λ_max_ = 410 nm, 1.0 OD) was observed, possibly because of Pb^2+^. A similar behavior was noticed in the case of G3, because of Cd^2+^. G5 exhibited a peak value of λ_max_ = 420 nm and 1.11 OD, because of the presence of both Cd^2+^ and Pb^2+^. The value did not shift much, possibly because of the presence of a metal mixture instead of single metal ions. Similarly, no prominent color change in the different groups was observed, in accordance with the optical absorbance measurements (Figure 7a,b). However, a slight change in color and absorbance in group 4 was observed, which consists of all metal ions except Cd^2+^ and Pb^2+^. This suggests that other metal ions also interfere with the nanoparticles. Therefore, G6 and G7 were included in the study, including all metal ions except Cd^2+^ and Pb^2+^ and either As^3+^ or Cr^3+^, respectively. No prominent color change was observed in G6 and G7. An increase in absorption intensity in G6 was observed, which was not the case for G7. This confirmed that As^3+^ slightly interferes in a metal ion mixture. After successful qualitative detection and development of a sensing probe, quantification of the detection limit for Cd^2+^ and Pb^2+^ using ʟ-car-AgNP1 was carried out, and LOD and LOQ were calculated. In Figure 7c and Figure 7e, only a single metal (i.e., Cd^2+^ or Pb^2+^) was added to ʟ-car-AgNP1, and the changes as functions of the metal ion concentration were measured. The changes in absorbance were plotted as functions of the concentrations of Cd^2+^ and Pb^2+^ (Figure 7d,f). The linear fit for Cd^2+^ showed an excellent *R*^2^ value of 0.9739 with LOD and LOQ values of 141.79 ppb (1.2 μM) and 238.56 ppb (2.12 μM), respectively. In the case of Pb^2+^, LOD and LOQ were found to be 131.33 ppb (0.63 μM) and 397.99 ppb (1.92 μM) with an *R*^2^ value of 0.9866. In Table 1, recent studies on the colorimetric detection of Cd^2+^ and Pb^2+^ using nanoparticles are summarized, along with their detection limits.

**Table 1:** Recent studies on sensitive colorimetric metal detection using various nanoparticles.

Nanoparticles	Metal detected	Detection method	LOD	Ref.

cysteine- and methionine-capped AgNPs	arsenic	paper-based colorimetric	0.0005 ppm	[33]
glutathione-capped AuNPs	arsenic	RGB colorimetric	0.12 ppb	[35]
citrate-capped AgNPs	arsenic	colorimetric	5.3 ppb	[36]
*Boswellia sacra* extract-stabilized AgNPs	arsenic	colorimetric	0.143 ppm	[37]
starch-stabilized AgNPs	chromium	colorimetric	0.93 μM	[38]
chlorophyll-coated AgNPs	chromium	colorimetric	0.62 μM	[39]
morin + PVP-capped AgNPs	chromium	colorimetric	4 × 10^−5^ M	[40]
secnidazole functionalized AgNPs	cadmium	colorimetric	0.07 μM	[41]
grape juice-stabilized AgNPs	cadmium	colorimetric	4.95 μM	[42]
*Annona muricata* extract-stabilized AuNPs	cadmium	colorimetric	4.45 × 10^−8^ M	[43]
orange peel extract-stabilized AuNPs	lead	colorimetric	13.31 μM	[44]
curcumin AgNPs	lead	colorimetric	13.6 μM	[45]
maltol-capped AgNPs	lead	colorimetric	0.067 μM	[46]
ʟ-carnosine-capped AgNP1	cadmium and lead	colorimetric	141.79 ppb (1.2 μM) and 131.33 ppb (0.63 μM)	this work
ʟ-carnosine-capped AgNP2	arsenic and chromium	colorimetric	215.35 ppb (2.8 μM) and 245.49 ppb (4.7 μM)	this work

### Interference study and quantification of the detection limit for As^3+^ and Cr^3+^ using ʟ-car-AgNP2

**Figure 8:**
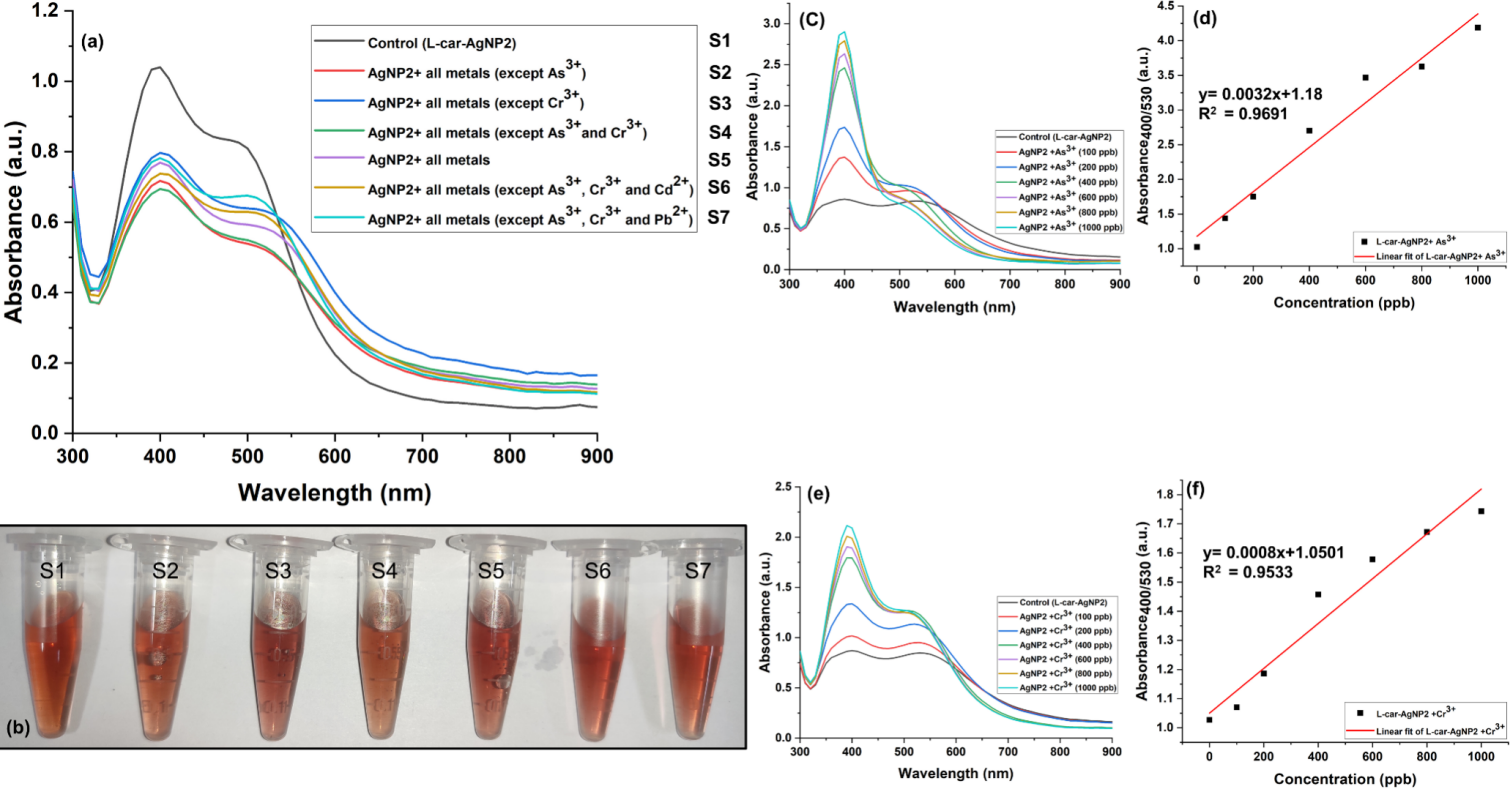
Interference study regarding the metal ion selectivity of ʟ-car-AgNP2. (a) Optical absorbance of different groups S2–7 and the control sample (S1). (b) Colorimetric observation of metal ion selectivity in the presence of groups S1–7. (c) UV–vis spectral absorbance of ʟ-car-AgNP2 with different As^3+^ concentrations (100–1000 ppb) and (d) the corresponding linear curve. (e) UV–vis spectral absorbance of ʟ-car-AgNP2 with different Cr^3+^ concentrations (100–1000 ppb) and (f) the corresponding linear curve.

The usability of ʟ-car-AgNP2 as a probe for the selective detection of As^3+^ and Cr^3+^ was assessed in an interference study, where several groups of metal mixtures were prepared (Figure 8). For the interference study, metal ions, namely, Al^3+^, Cd^2+^ Zn^2+^, Hg^2+^, Ni^2+^, Cu^2+^, Pb^2+^, Mo^2+^, and Fe^3+^, were mixed in a volume of 1 mL with a final concentration of 0.5 ppm for the metal ions. The metal ion mixtures were grouped as sample groups S1–7, where S1 included no metal ions (control group). S2 and S3 included all metal ions except either As^3+^ or Cr^3+^, respectively. S4 included all metal ions except both As^3+^ and Cr^3+^. S5 contained all metal ions, including As^3+^ and Cr^3+^. After adequately mixing the metal ions, ʟ-car-AgNP2 was dispersed into the test system and underwent colorimetric and spectrophotometric investigations. The slight color change observed in S2, S3, and S5 indicated the successful detection of As^3+^ and Cr^3+^, even in the presence of other metal ions. The colorimetric detection was further validated through optical spectrometry, which showed increased absorbance (Figure 8a) in S2 (λ_max_ = 515 nm, 0.52 OD), S3 (λ_max_ = 530 nm, 0.62 OD), and S5 (λ_max_ = 530 nm, 0.56 OD) compared to S1 (control, λ_max_ = 500 nm, 0.8 OD). However, a slight change in color and plasmon peak was observed in S4 (λ_max_ = 510 nm, 0.53 OD) (Figure 8b). This suggested that other metals also interfere with the nanoparticles. Therefore, S6 (λ_max_ = 505 nm, 0.62 OD) and S7 (λ_max_ = 500 nm, 0.67 OD) were included in the study, which included of all metal ions except As^3+^ and Cr^3+^ and either Cd^2+^ or Pb^2+^, respectively. However, no prominent color change was observed in S6 and S7, but an increase in the plasmon peak intensity in S6, but not in S7. This confirmed that Cd^2+^ slightly interferes with detecting As^3+^ and Cr^3+^. This is probably due to the interaction of ʟ-car-AgNPs with bivalent metal ions, as reported elsewhere [19].

After successful qualitative detection and the development of a sensing probe, quantification of the detection limit for As^3+^ and Cr^3+^ using ʟ-car-AgNP2 was carried out, and LOD and LOQ were calculated. In Figure 8c and Figure 8e, only a single metal (i.e., As^3+^ or Cr^3+^, respectively) was added to ʟ-car-AgNP2, and the changes as functions of the metal ion concentration were measured. The changes in absorbance were plotted as functions of the concentrations of As^3+^ and Cr^3+^ (Figure 8d,f). The linear fit for As^3+^ showed an *R*^2^ value of 0.9691 with LOD and LOQ values of 215.35 ppb (2.8 μM) and 591.42 ppb (7.8 µM), respectively. In the case of Cr^3+^, the obtained values of LOD and LOQ were 245.49 ppb (4.7 μM) and 743.92 ppb (14.2 μM), respectively, with an *R*^2^ value of 0.9533. In Table 1, recent studies on the colorimetric detection of As^3+^ and Cr^3+^ using nanoparticles and their detection limits are summarized.

### Catalytic potential of ʟ-car-AgNPs in degradation of *p*-nitrophenol

**Figure 9:**
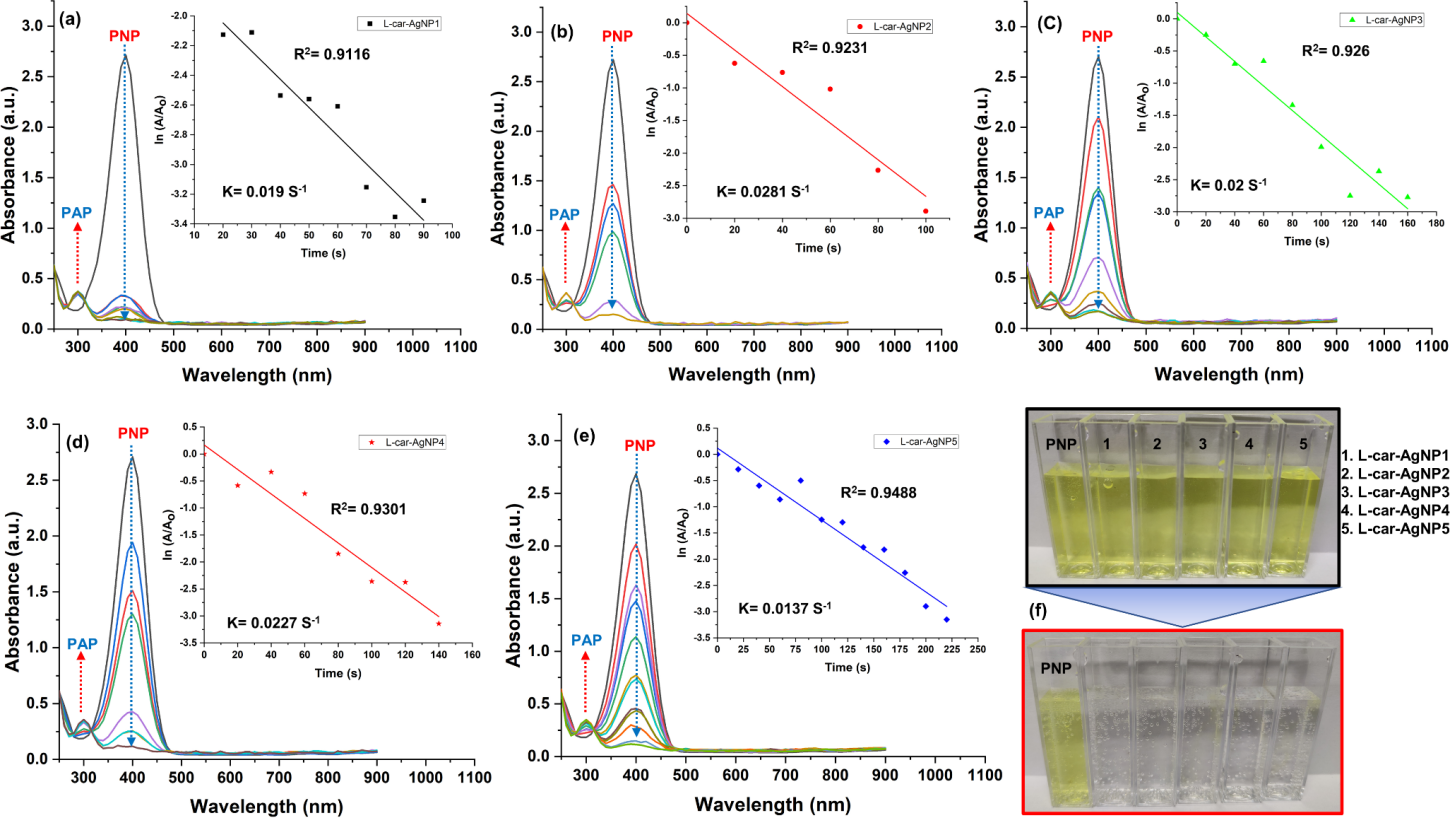
(a–e) UV–vis absorption measurements showing the catalytic degradation of 0.25 mM P-NP using ʟ-carnosine-capped AgNP1–5. The inset graphs show pseudo-first-order kinetics for the degradation of P-NP. (f) Color change in P-NP solutions before and after the addition of ʟ-car-AgNPs. The dashed red and blue arrows indicate the formation of P-AP and the degradation of P-NP, respectively.

The catalytic efficacy of ʟ-car-AgNPs was evaluated using the degradation of P-NP and the formation of P-AP as a model reaction. This transformation, catalyzed by noble metal nanoparticles in the presence of NaBH_4_ as reducing agent, is widely utilized to assess the catalytic performance of nanomaterials [14]. All catalytic experiments were performed in a standard quartz cuvette, and the spectral changes were monitored as the reaction progressed. The reaction mixture consisted of an aqueous solution of P-NP (0.25 and 1 mM) and 0.1 M NaBH_4_ to which ʟ-car-AgNPs were added. It was noticed that, upon introducing NaBH_4_, the light yellow color changes to intense yellow-green, accompanied by an absorption shift from 317 to 400 nm, indicating the formation of *p*-nitrophenolate ions (an intermediate) in the basic reaction medium [47]. These intermediate ions were further degraded in the presence of ʟ-car-AgNPs as a nanocatalyst to the stable product P-AP. The conversion of P-NP to P-AP was monitored through UV–vis spectroscopy. In UV–vis measurements, there was a decrease in absorption intensity at 400 nm, and a minor peak around 300 nm was observed due to the formation of P-AP (Figure 9).

Figure 9 illustrates the P-NP degradation at 0.25 mM using ʟ-car-AgNPs (1 ± 0.2 OD). ʟ-car-AgNP1 led to the degradation of 0.25 mM P-NP within 90 s of the reaction, achieving a degradation efficiency of 95.5% (Figure 9a). In comparison, ʟ-car-AgNP2, ʟ-car-AgNP3, ʟ-car-AgNP4, and ʟ-car-AgNP5 achieved complete P-NP degradation within 100, 160, 140, and 220 s, respectively, with catalytic efficiencies of 96.18%, 96.52%, 94.61% and 95.71% (Figure 9b–e). The color change from yellow to colorless indicates complete degradation of P-NP and, thus, the catalytic potential of the nanoparticles (Figure 9f). Increasing the P-NP concentration to 1 mM required longer times until full degradation, as documented in Supporting Information File 1 (Figure S6). The degradation times for 1 mM P-NP using ʟ-car-AgNP1, ʟ-car-AgNP2, ʟ-car-AgNP3, ʟ-car-AgNP4, and ʟ-car-AgNP5 were 270, 330, 300, 270, and 420 s, respectively (Supporting Information File 1, Figure S6). The different degradation times might be attributed to differences in particle sizes and monodispersity. TEM and DLS measurements revealed that ʟ-car-AgNP1 nanoparticles were spherical and monodisperse, whereas the other nanoparticles (ʟ-car-AgNP2–5) exhibited polydispersity and non-spherical shapes. This is in accordance with results from Jiji et al., where different shapes of gold nanoparticles had varying efficiencies in the catalytic degradation of nitrophenol compounds, with spherical nanoparticles being highly effective [48]. Kinetic analysis was performed based on pseudo-first-order reaction kinetics for the concentration of 0.25 mM P-NP. The spectral absorbance at a concentration of 1 mM P-NP exceeded 4 OD, necessitating a dilution factor of 10 to achieve an observable OD of 1.2 (Supporting Information File 1, Figure S6). Given the excess NaBH_4_ used in the reaction, the borohydride concentration was assumed to remain constant. The rate constants (*k*) were computed from the linear plots of ln(*A*/*A*_0_) as a function of time, where *A* and *A*_0_ denote the absorbances at time *t* and *t* = 0, respectively. The calculated rate constants were 0.019, 0.028, 0.020, 0.023, and 0.014 s^−1^ for ʟ-car-AgNP1, ʟ-car-AgNP2, ʟ-car-AgNP3, ʟ-car-AgNP4, and ʟ-car-AgNP5 respectively, at 25 ± 2 °C. This suggests that the catalytic activity of the nanoparticles is in the order of AgNP5 < AgNP1 < AgNP3 < AgNP4 < AgNP2. Interestingly, the catalytic activity of ʟ-car-AgNPs does not depend on the hydrodynamic size of the nanoparticles obtained from DLS. These results confirmed that the catalytic activity of metal nanoparticles does not necessarily increase with decreasing size, potentially because of the strong curvature of the surface of very small particles, which has also been reported [14,49]. In addition, the obtained rate constants for ʟ-car-AgNP4, ʟ-car-AgNP3, and ʟ-car-AgNP2 are nearly the same, but the lower rate constant in case of AgNP5 might be due to the formation of larger aggregates, which was confirmed by optical spectroscopy and TEM analysis. The high efficiency of AgNP2 might be due to its quasi-spherical shape.

The catalytic degradation of P-NP by ʟ-car-AgNPs can be attributed to the unique optical and catalytic properties of AgNPs, which are enhanced by their high surface-to-volume ratio and plasmonic effects. The observed pseudo-first-order kinetics suggest that the rate-determining step involves the adsorption of P-NP onto the nanoparticle surface, followed by the electron transfer from NaBH_4_ to the adsorbed P-NP molecules facilitated by the AgNPs. The obtained rate constants indicate that ʟ-carnosine-capped AgNPs are comparable to or more efficient than other noble metal nanoparticles (Table 2), underscoring their potential as cost-effective catalysts for the degradation of nitroaromatic compounds, which are prevalent in industrial effluents and pose significant environmental risks.

**Table 2:** Comparative study of the degradation of P-NP using various nanoparticles.

Nanoparticles	Time of degradation (min)	Catalyst concentration	P-NP concentration	Ref.

*Acacia nilotica* (stem)-stabilized AgNPs	10	5 mg	0.1 mM	[50]
citrate-capped Au- NPs	14	200 µL	0.2 mM	[51]
citrate-capped AgNPs	120	0.54 mg	0.1 mM	[52]
cobalt (Co)-doped ZnO NPs	180	30 mg	30 mg/L (30 ppm)	[53]
cobalt oxide nanocomposites	15	200 µL (1 mg/mL)	9.03 mg/L (9.030 ppm)	[54]
ʟ-car-AgNPs	<5	50 µL (1 ± 0.2 OD)	0.25 mM (34.75 ppm)	this work
	<10		1 mM (139 ppm)	

## Conclusion

Silver nanoparticles with tunable absorption bands were synthesized for the first time using ʟ-carnosine as a capping agent. This novel approach has facilitated the development of nanoparticles that exhibit controlled size and stability, which is crucial for various applications. ʟ-carnosine-capped silver nanoparticles, designated as ʟ-car-AgNPs, were extensively characterized using UV–vis absorbance, DLS, zeta potential, FTIR, Raman spectroscopy, TEM, SEM-EDX, and XRD. This comprehensive analysis elucidates the optical absorbance, sizes, surface charges, functional group interactions, elemental compositions, and diffraction patterns of ʟ-car-AgNPs. Furthermore, different sizes of ʟ-car-AgNPs (ʟ-car-AgNP1 to ʟ-car-AgNP5) were explored regarding their potential as metal ion sensors. Among them, ʟ-car-AgNP1 showed selective detection of Cd^2+^ and Pb^2+^. In contrast, ʟ-car-AgNP2, ʟ-car-AgNP3, and ʟ-car-AgNP4 showed affinity towards As^3+^ and Cr^3+^ detection. Interestingly, ʟ-car-AgNP5 did not detect any metal ions. The quantitative limits of detection (LODs) of Cd^2+^, Pb^2+^, As^3+^, and Cr^3+^ were 141.79, 131.33, 215.35, and 245.49 ppb, respectively. These values suggest a competitive sensitivity compared to other nanoparticle-based sensors reported in the literature.

Additionally, the catalytic activity of ʟ-car-AgNPs was studied regarding the degradation of 0.25 and 1 mM concentrations of *p*-nitrophenol (P-NP) within 5 and 10 min, respectively. The catalytic efficiencies for 0.25 mM P-NP were 95.5%, 96.18%, 96.52%, 94.61%, and 95.71% achieved with ʟ-car-AgNP1, ʟ-car-AgNP2, ʟ-car-AgNP3, ʟ-car-AgNP4, and ʟ-car-AgNP5, respectively. The rapid degradation demonstrates the catalytic prowess of ʟ-car-AgNPs and highlights their potential in environmental remediation applications. The successful synthesis of ʟ-car-AgNPs with tunable plasmon resonance has paved the way for their application as colorimetric sensors for heavy metal detection and as efficient catalysts for the degradation of nitroaromatic compounds. The selectivity regarding metal ions based on the nanoparticle size demonstrates the potential for developing tailored sensors for specific applications. The catalytic activity of the nanoparticles in the degradation of *p*-nitrophenol highlights their potential as cost-effective and environmentally friendly catalysts for the remediation of industrial effluents. Future studies will focus on optimizing these nanoparticles for broader applications and understanding their interaction mechanisms at the molecular level.

## Supporting Information

The file contains six figures. Figures S1 and S2 show hydrodynamic sizes and zeta potentials obtained from DLS measurements. Figures S3 and S4 show optimized geometries of ʟ-carnosine and the ʟ-carnosine–AgNPs complex, respectively. Figure S5 shows the colorimetric and spectrophotometric detection of metal ions using ʟ-car-AgNP3 and ʟ-car-AgNP4. Figure S6 shows the catalytic degradation of 1 mM *p*-nitrophenol using ʟ-car-AgNPs.

File 1 Additional figures.

## Data Availability

All data that supports the findings of this study is available in the published article and/or the supporting information of this article.
