# Supplementary material for: Facile synthesis of size-tunable L-carnosine-capped silver nanoparticles and their role in metal ion sensing and catalytic degradation of p-nitrophenol
Source: Beilstein J Nanotechnol. 2024 Dec 6;15:1576–92. doi: 10.3762/bjnano.15.124 (PMC11635285; doi:10.3762/bjnano.15.124)
Supplement: File 1 — Additional figures. [file Beilstein_J_Nanotechnol-15-1576-s001.pdf]

BEILSTEIN JOURNAL OF NANOTECHNOLOGY

Supporting Information

for

Facile synthesis of size-tunable L-carnosine-capped silver nanoparticles and their role in metal ion sensing and catalytic degradation of *p*-nitrophenol

Akash Kumar, Ridhima Chadha, Abhishek Das, Nandita Maiti and Rayavarapu Raja Gopal

Beilstein J. Nanotechnol. **2024,** *15,* 1576–1592. doi:10.3762/bjnano.15.124

Additional figures

License and Terms: This is a supporting information file under the terms of the Creative Commons Attribution License (https://creativecommons.org/licenses/by/4.0). Please note that the reuse, redistribution and reproduction in particular requires that the author(s) and source are credited and that individual graphics may be subject to special legal provisions.
The license is subject to the *Beilstein Journal of Nanotechnology* terms and conditions: (https://www.beilstein-journals.org/bjnano/terms)

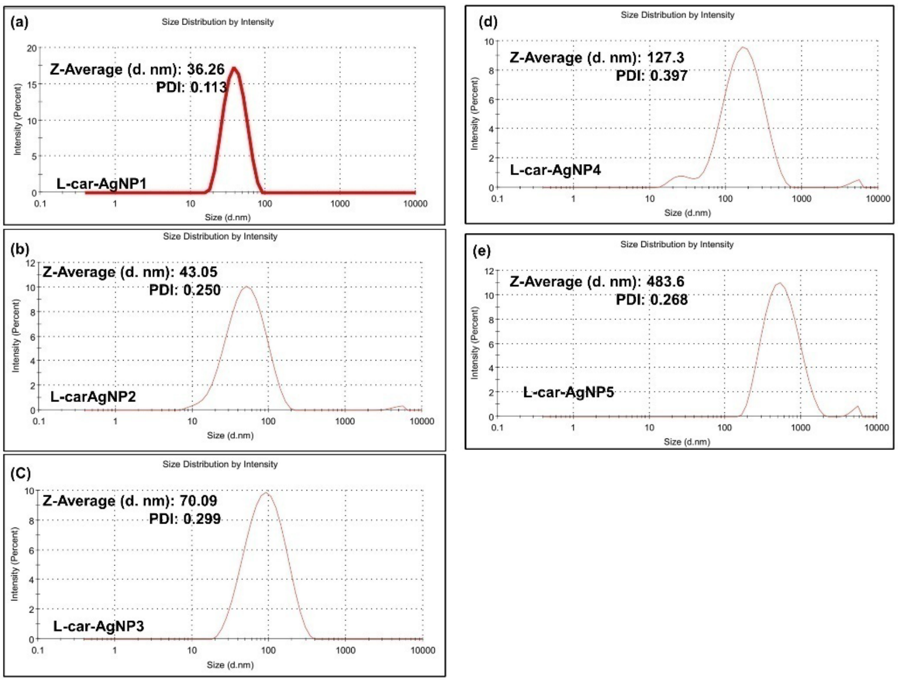

Figure S1: Hydrodynamic size of (a) L-car-AgNP1, (b) L-car-AgNP2, (c) L-car-AgNP3, (d) L-car-AgNP4, and (e) L-car-AgNP5.

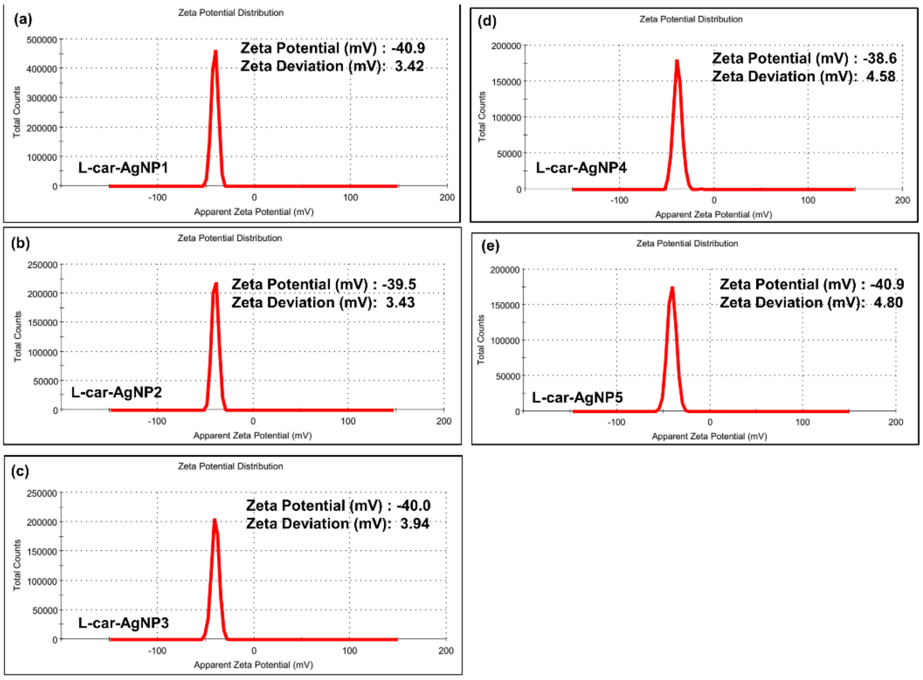

Figure S2: Zeta potential of (a) L-car-AgNP1, (b) L-car-AgNP2, (c) L-car-AgNP3, (d) L-car-AgNP4, and (e) L-car-AgNP5.

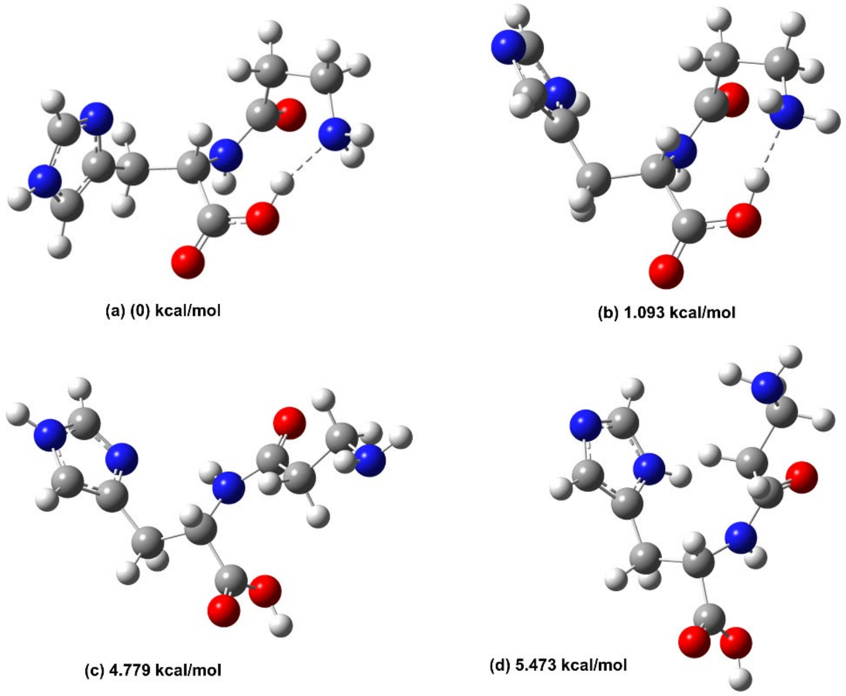

Figure S3: Optimized geometries of L-carnosine along with relative stabilization energies. The color codes to identify the atoms are N (blue), O (red), C (grey), and H (white).

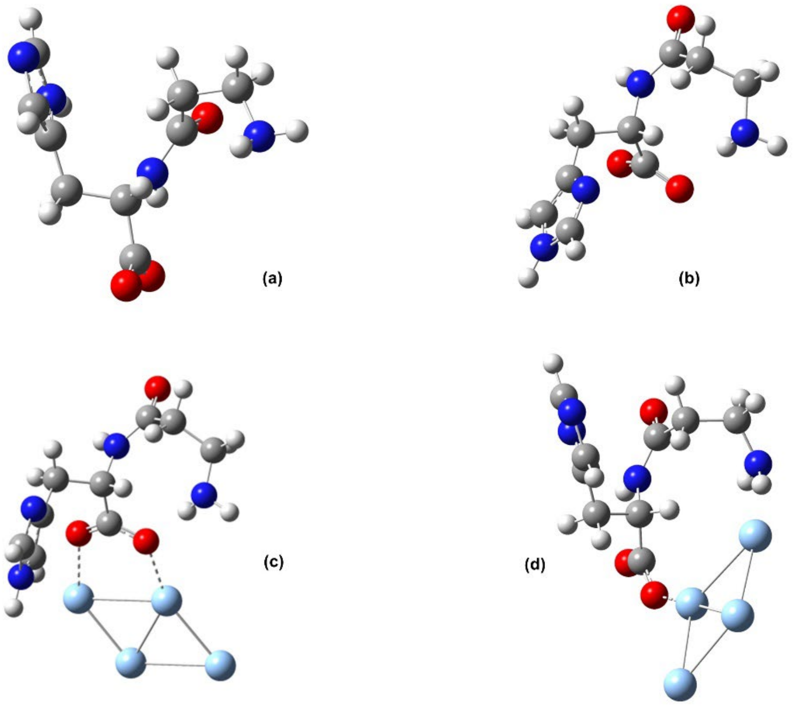

Figure S4: Optimized geometries of L-carnosine–AgNPs complex. The color codes to identify the atoms are N (blue), O (red), C (grey), H (white), and Ag (light blue).

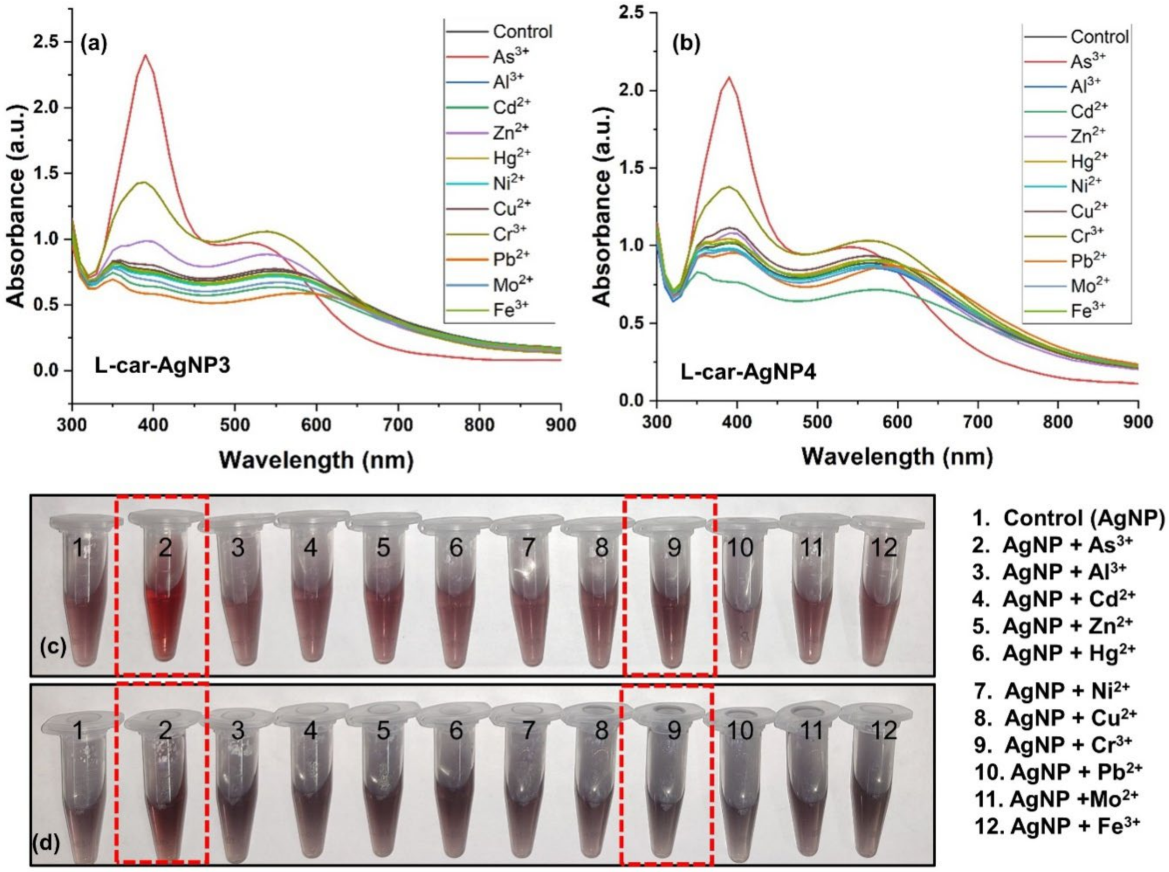

Figure S5: Spectrophotometric detection of heavy metal ions using (a) L-car-AgNP3 and (b) L-car-AgNP4. Colorimetric detection of heavy metal ions using (c) L-car-AgNP3 and (d) L-car-AgNP4.

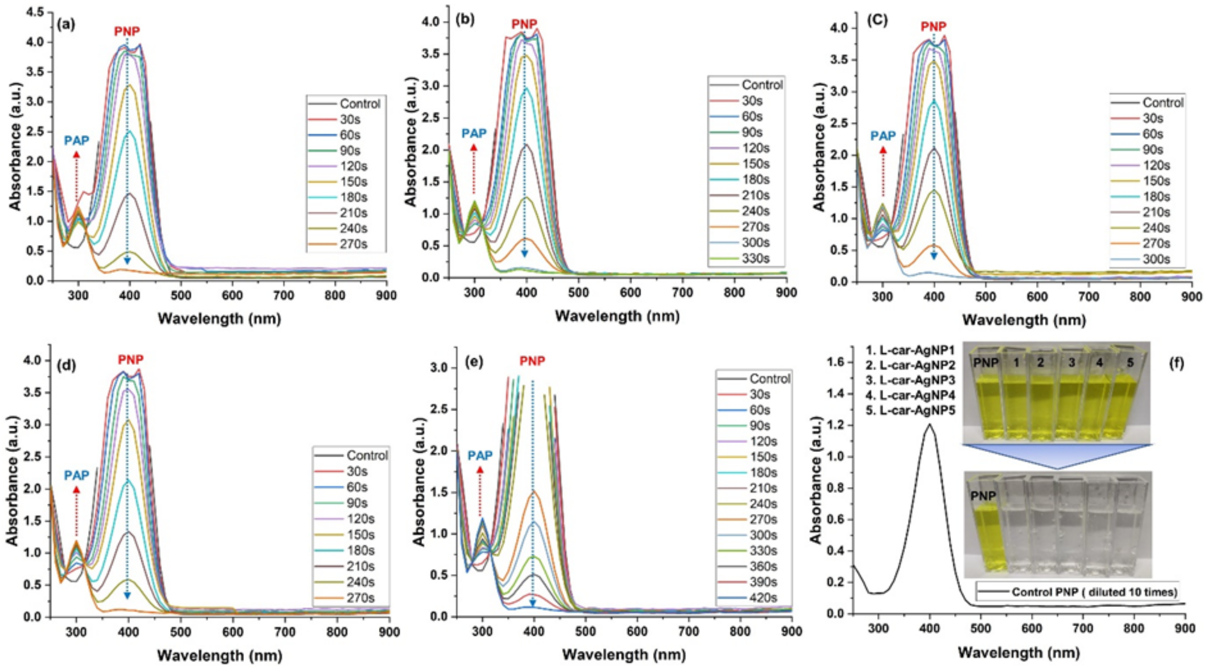

Figure S6: Catalytic degradation of 1 mM P-NP using (a) L-car-AgNP1, (b) L-car-AgNP2, (c) L-car-AgNP3, (d) L-car-AgNP4, and (e) L-car-AgNP5. (f) Colorimetric image showing the degradation of P-NP in the presence of L-car-AgNPs.
